# Tumor necrosis factor α triggers proliferation of adult neural stem cells via IKK/NF-κB signaling

**DOI:** 10.1186/1471-2202-7-64

**Published:** 2006-09-20

**Authors:** Darius Widera, Ilja Mikenberg, Margitta Elvers, Christian Kaltschmidt, Barbara Kaltschmidt

**Affiliations:** 1 University of Witten/Herdecke, Stockumer Str. 10, 58448 Witten, Germany; 2 Vascular Biology, Rudolf Virchow Center, DFG Research Center for Experimental Biomedicine, Versbacher Str. 9, 97078 Würzburg, Germany

## Abstract

**Background:**

Brain inflammation has been recognized as a complex phenomenon with numerous related aspects. In addition to the very well-described neurodegenerative effect of inflammation, several studies suggest that inflammatory signals exert a potentially positive influence on neural stem cell proliferation, migration and differentiation. Tumor necrosis factor alpha (TNF-α) is one of the best-characterized mediators of inflammation. To date, conclusions about the action of TNF on neural stem or progenitor cells (NSCs, NPCs) have been conflicting. TNF seems to activate NSC proliferation and to inhibit their differentiation into NPCs. The purpose of the present study was to analyze the molecular signal transduction mechanisms induced by TNF and resulting in NSC proliferation.

**Results:**

Here we describe for the first time the TNF-mediated signal transduction cascade in neural stem cells (NSCs) that results in increased proliferation. Moreover, we demonstrate IKK-α/β-dependent proliferation and markedly up-regulated cyclin D1 expression after TNF treatment. The significant increase in proliferation in TNF-treated cells was indicated by increased neurosphere volume, increased bromodeoxyuridin (BrdU) incorporation and a higher total cell number. Furthermore, TNF strongly activated nuclear factor-kappa B (NF-κB) as measured by reporter gene assays and by an activity-specific antibody. Proliferation of control and TNF-treated NSCs was strongly inhibited by expression of the NF-κB super-repressor IκB-AA1. Pharmacological blockade of IκB ubiquitin ligase activity led to comparable decreases in NF-κB activity and proliferation. In addition, IKK-β gene product knock-down via siRNA led to diminished NF-κB activity, attenuated cyclin D1 expression and finally decreased proliferation. In contrast, TGFβ-activated kinase 1 (TAK-1) is partially dispensable for TNF-mediated and endogenous proliferation. Understanding stem cell proliferation is crucial for future regenerative and anti-tumor medicine.

**Conclusion:**

TNF-mediated activation of IKK-β resulted in activation of NF-κB and was followed by up-regulation of the bona-fide target gene cyclin D1. Activation of the canonical NF-κB pathway resulted in strongly increased proliferation of NSCs.

## Background

During mammalian central nervous system (CNS) development, multipotent precursor cells (stem cells) undergo division, cell fate specification, and maturation in response to extrinsic cues. These neural stem cells are characterized by the ability to undergo cell division and to differentiate into multiple cell types, e.g. neurons or glial cells.

There are two major sources of adult neural stem cells within the adult brain: the subgranular zone of the hippocampus and the subventricular zone (SVZ) [1,2]. SVZ-derived NSCs can be cultured as self-adherent cell clusters called neurospheres [2]. Such 3D neurospheres can be kept in culture for several passages without losing their proliferation, migration and differentiation capabilities.

Until the 1990s, all studies of neural stem cell proliferation were limited to examining the proliferation of precursors in embryonic tissue. Recently, several isolation and culture protocols have been established that have enabled proliferation to be studied in cultured adult neural stem cells [3-6]. It is noteworthy that under normal conditions, proliferation (division) is tightly controlled. Cytokine-induced cell death and dysfunction play an important role in the pathogenesis of a variety of disease conditions, including brain inflammation. However, cytokine production within the adult brain is strongly up-regulated by inflammation. This response has been well described in demyelinating diseases, e.g. multiple sclerosis, experimental autoimmune encephalomyelitis, viral or bacterial infection, trauma and ischemia [7]. Much of the inflammatory signal transduction can be considered as an innate immune response triggered by tumor necrosis factor (TNF), one of the crucial inflammation mediators [8,9]. As a model for brain inflammation, we initially investigated the transcriptional profile of TNF-treated astroglioma cells [10]. We demonstrated more than 800 TNF-regulated genes. Macrophage Chemoattractant Protein 1 (MCP-1) was strongly up-regulated and secreted into the medium.

It is well established that neural stem cells express various chemokine receptors as a result of brain pathology (see [11] and [12]). In addition to MCP-1, expression of stromal derived factor 1 (SDF1), stem cell factor (SCF) and vascular endothelial growth factor (VEGF) has been reported. In subsequent experiments, we therefore tested the possibility that MCP-1 induces NSC migration [13] and found a significant effect.

In view of the very well-described TNF secretion during inflammatory diseases and the very potent induction of NSC migration by MCP-1, we hypothesized that in pathological situations these cells migrate from the SVZ to the area of the lesion. This hypothesis accords with a model proposed by Muller et al. [11]. According to this model, neural stem cells are attracted by inflammation, reactive astrocytosis and angiogenesis. Thus, NSCs are exposed after migration to TNF at the area of inflammation.

In the present study, we analyzed the biological effect and signal transduction pathway of TNF in NSCs *in vitro*. The advantage of the *in vitro *approach is a biochemically defined environment with minimal risk of unwanted cross-activation by cytokines and/or unknown *in vivo *cell-cell interactions.

Within the nervous system, TNF (a 17 kDa protein) binds to TNF receptors (TNF-Rs) expressed on both glia and neurons [14]. Expression of the TNF-α gene is subject to auto-regulation via activated NF-κB [15]. Two different receptors have been identified: p55 (TNF-RI) and p75 (TNF-RII). The p55 receptor plays the major role in NF-κB activation [16]. Furthermore, it has been shown that the IKK-α/β-complex is crucial for TNF-mediated NF-κB activation [17], and there is evidence that TGFβ activated kinase-1 (TAK-1) is involved in the TNF-induced signaling cascade [18].

Cyclin dependent kinase 4 and 6 (CDK4/6) signaling is crucial in neural stem cell cycle regulation [19]. Moreover, formation of a complex between CDK 4 and cyclin D1 is necessary for NSC cell cycle progression by promoting passage through the G1/S restriction point. In contrast, Burgess et al. showed clearly that CDK4/cyclin D3 in tumor cells is critical for G2/M progression and the fidelity of mitosis [20]. Kaltschmidt et al. described cyclin D1-dependent proliferation of HeLa cells, which was abrogated by over-expression of IκB [21]. Guttridge et al. were the first to show that NF-κB controls proliferation through transcriptional regulation of cyclin D1 in embryonic fibroblasts [22].

In this study, we describe for the first time the TNF-mediated signal cascade in adult neural stem cells. TNF treatment leads to strong IKK-mediated NF-κB activation and up-regulation of cyclin D1 expression, resulting in increased proliferation. Taken together, these results show that NF-κB has a crucial role in the proliferation of NSCs induced by inflammation.

## Results

### Characterization of neural stem cells derived from the adult subventricular zone

**Figure 1:**
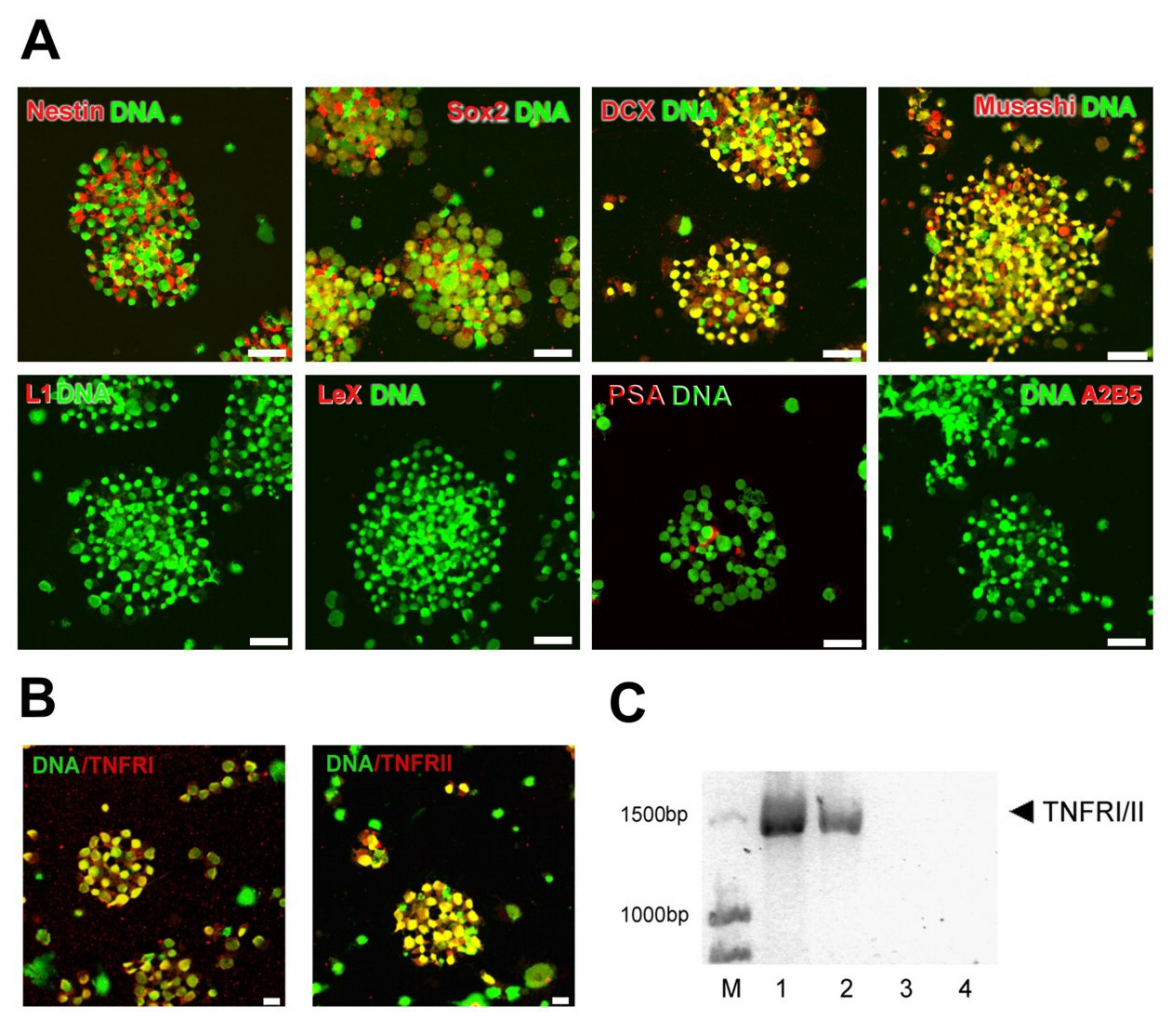
**Expression pattern of cultured, rat derived neurospheres**. (A)Neurospheres were collected on glass slides by cytospin centrifugation, fixed and stained for Nestin, Sox2, DCX, Musashi, L1, LeX, PSA-NCAM and A2B5. DNA was stained using SYTOX (green). Most of the cells analyzed were positive for Nestin, Sox2, DCX, Musashi, Notch-1 (data not shown) and Notch-2 (data not shown) and negative for L1, LeX and the differentiation marker A2B5 for oligodendrocyte progenitors. Moreover, we detected moderate expression of PSA-NCAM, a marker for migrating neuronal precursor cells. Bar, 50 μm. (B) Neurospheres express both TNF-receptors. Neurospheres were collected, fixed and stained for TNFR-I and TNFR-II. Nuclei were stained with SYTOX (green). Note the high expression of both TNFR-I and II. (C) RT-PCR confirms expression of both TNF receptors. M: Marker, lane 1: TNFR-I, lane 2: TNFR-II. Lanes 3 and 4 are negative PCR controls without template.

The generation of neural stem/progenitor cells isolated from the subventricular zone was studied using specific stem-cell markers such as Nestin, Sox2, doublecortin and Musashi (see Fig. 1A). Most of the NSCs were Nestin-immunoreactive, a characteristic of neural stem cells, and they expressed the neural stem cell-specific transcription factor SOX2 [23]. Furthermore, they expressed doublecortin (DCX), a marker for newborn immature neurons that is also detected in proliferating neural cells [24], and the RNA-binding protein Musashi, which is essential for neurosphere formation and proliferation [25]. The population markers L1 and LeX, and the oligodendrocytic lineage marker A2B5, were not expressed. Moreover, we detected moderate expression of PSA-NCAM, a marker for migrating neuronal precursor cells. The differentiation markers β-III-tubulin (for neurons) and GFAP (for glial cells) were not expressed (data not shown). In order to analyze the effect of TNF on the NSCs, we investigated the expression of the specific TNF receptors I and II. Immunocytochemical staining showed that both TNF-RI and II are expressed on the cell surfaces (Fig. 1B). The intracellular localization of the TNF receptors (especially TNF-RII) is consistent with the recent finding that TNF signalosomes are endosome-localized [26]. We also verified the expression of TNF-RI and II by RT-PCR (see Fig. 1C).

### TNF induces an increase in neurosphere volume

**Figure 2:**
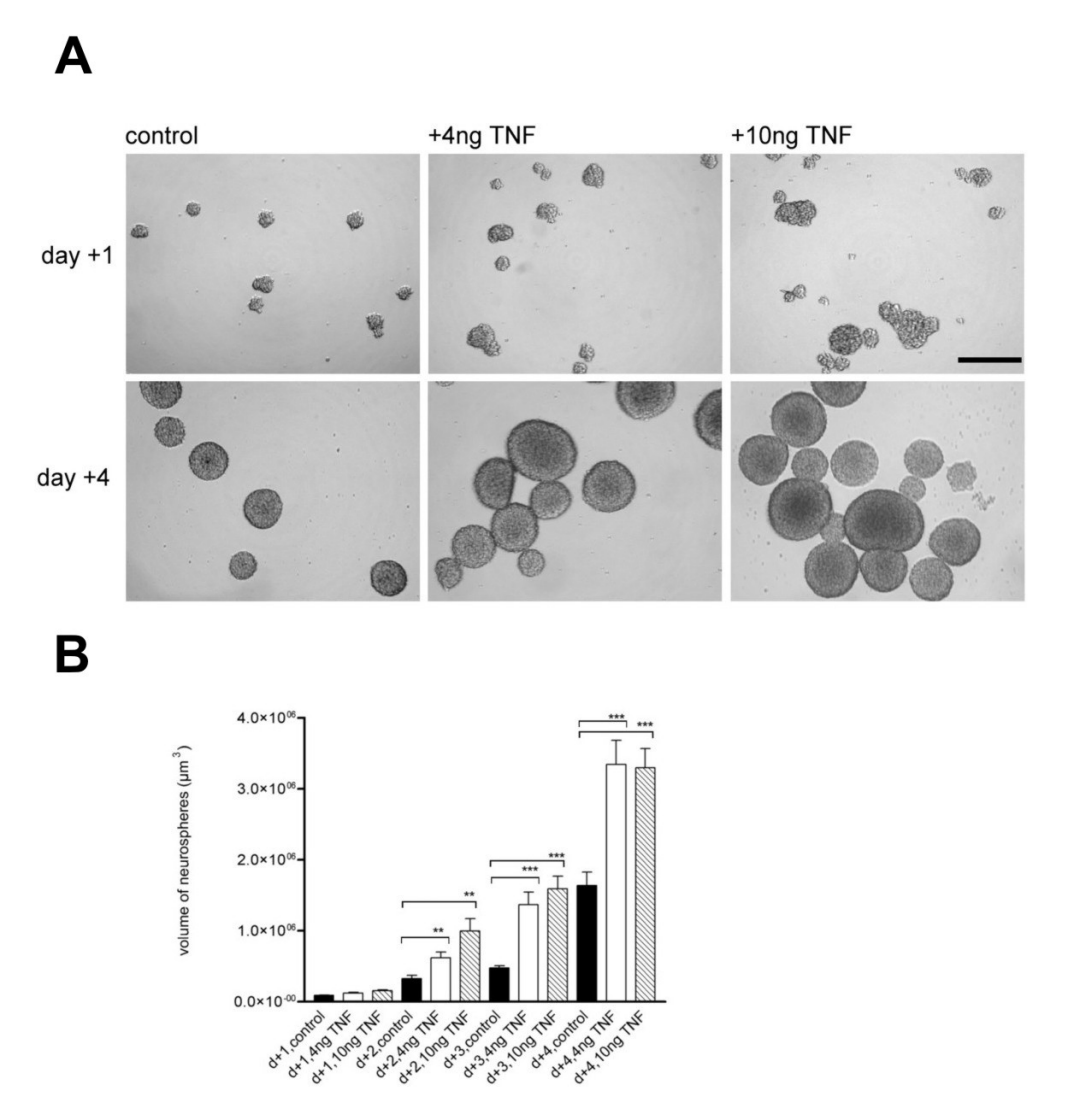
**TNF-α enhances growth of rat derived neurospheres**. (A) Photographs of neurospheres, 1 (upper panel) and 4 (lower panel) days after plating, show larger neurospheres after TNF treatment than in control neurospheres. Note that 10 ng/ml TNF has the greatest size induction effect. Bar, 200 μm. (B) Quantification of neurosphere volume. TNF-treated neurospheres show significantly increased volume compared to untreated neurospheres (*** P < 0.0001, **P < 0.05).

We used sub-cultured secondary neurospheres from passage 10 onwards. NSCs were treated for up to 4 days with TNF in different concentrations (4 ng/ml and 10 ng/ml). This resulted in a significant, dose-dependent increase in volume of the 3D neurospheres (Fig. 2A, B). We then asked whether the increased neurosphere volume results from enhanced NSC proliferation.

### Growth of neurospheres correlates with proliferation of NSCs

**Figure 3:**
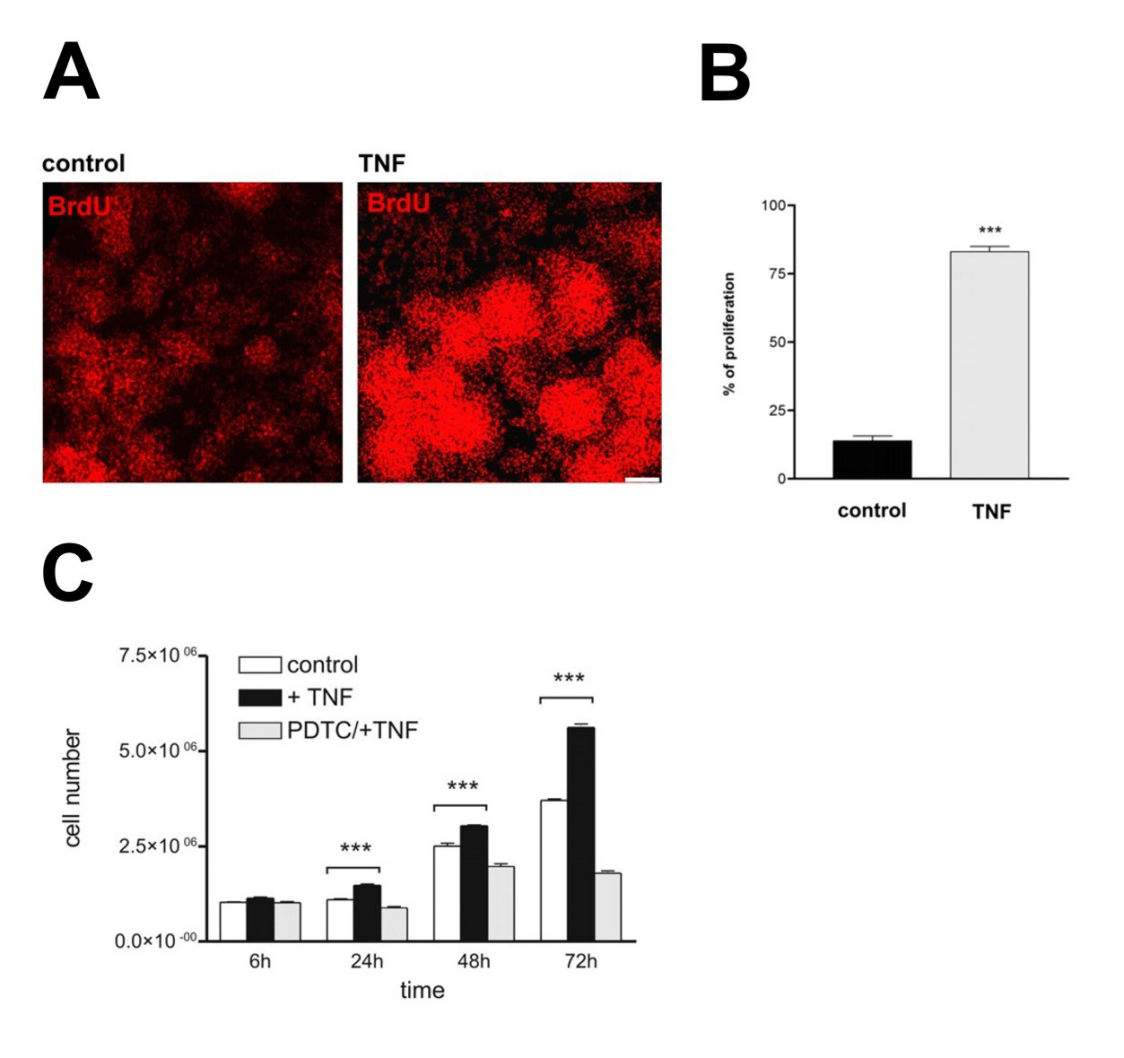
**Proliferation of neural stem cells is greatly enhanced by TNF treatment**. (A) Incorporation of bromodeoxyuridin (BrdU) into newly-synthesized DNA was determined using BrdU-dependent fluorescence enhancement of TO-PRO-3 (red). Cultures (+TNF) were treated with 10 ng/ml TNF for two days. Scale bar depicts 100 μm. Note the larger neurospheres after TNF treatment. (B) Quantification of BrdU incorporation is shown as mean ± SEM of at least four independent determinations (***P < 0.001). (C) Total cell number determination confirms higher proliferation of TNF-treated cells. Note that pharmacological blockade of the NF-κB pathway by PDTC counteracts TNF-induced proliferation (***P < 0.001).

We analyzed stem cell proliferation by BrdU incorporation. This technique has been extensively used to measure DNA replication during proliferation of mammalian cells. Incorporation of BrdU was measured in control NSC cultures and in cultures treated with 10 ng/ml TNF for 72 h (Fig. 3A).

**Figure 4:**
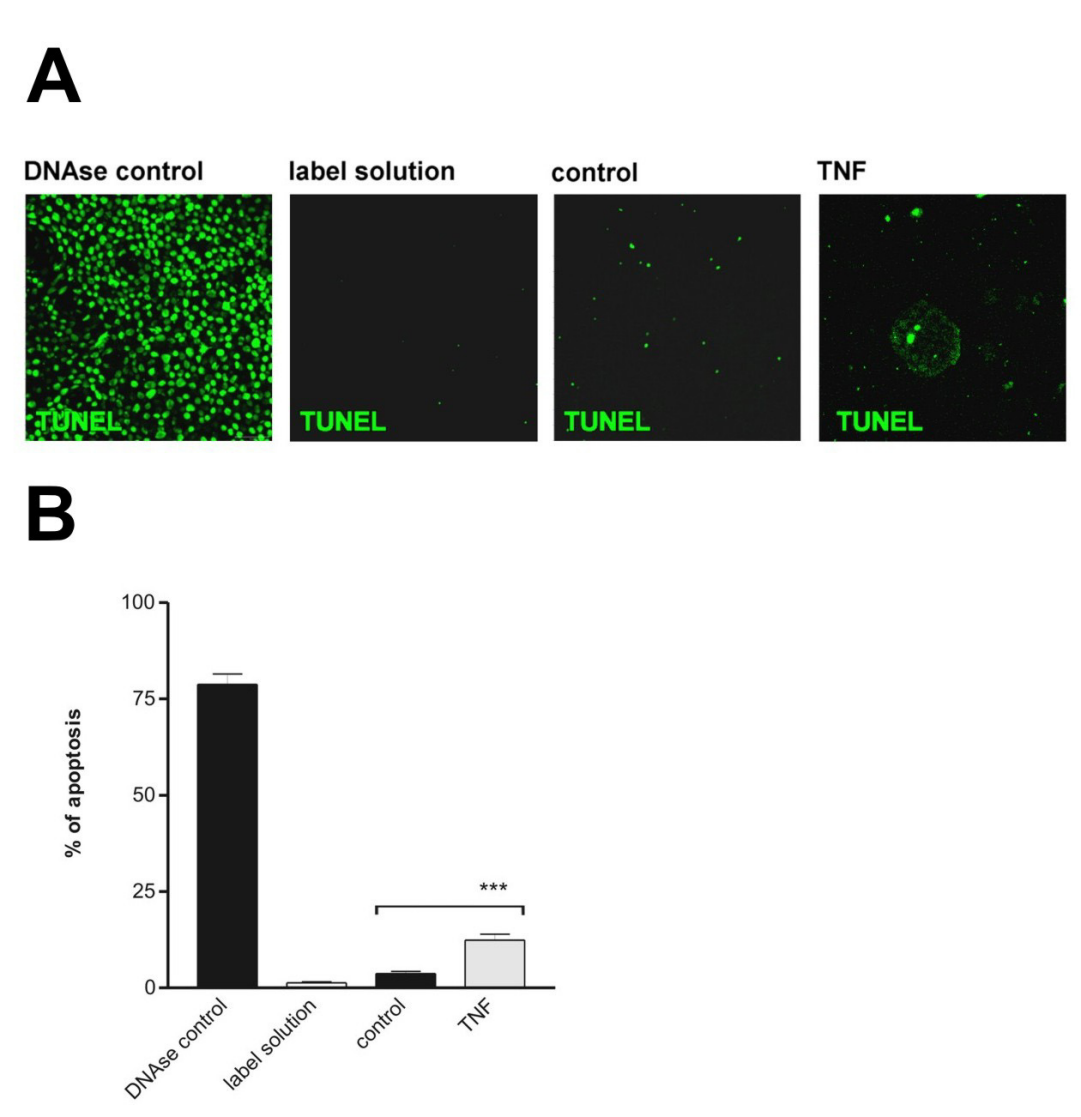
**TNF rarely triggers apoptosis in NSCs**. (A) NSCs were collected using cytospin centrifugation. Apoptosis was detected using TUNEL with FITC-conjugated dUTP. Control cultures were treated with DNAse I to generate nicked DNA. Terminal deoxynucleotidyl transferase was omitted for negative controls. TNF treatment only slightly increased the number of FITC-positive cells. (B) Quantification of apoptotic cells (***P < 0.01).

As expected, smaller spheres with lightly-labeled nuclei were seen in control cultures owing to endogenous proliferation. In contrast, TNF-treated neurospheres showed strong BrdU incorporation, an indication of rapidly proliferating cells. This might explain the marked increase in neurosphere volume after 72 h of TNF treatment (see Fig. 4A). Thus, TNF significantly (p < 0.001) activated NSC proliferation (Fig. 3B). BrdU incorporation was more than 6-fold higher in TNF-treated cells than in untreated controls.

Cell counting was also used as an independent measure of cell proliferation. TNF treatment significantly increased the cell number (see Fig. 3C). This effect disappeared when the cells were pre-incubated with IκB ubiquitin ligase activity inhibitor PDTC. This is a first hint that NF-κB is involved in TNF-mediated signaling (see also Fig. 9) in adult NSCs.

### TNF activates apoptosis only moderately in neural stem cells

Since one of the known physiological effects of TNF is the induction of apoptosis, we analyzed the frequency of apoptotic cells in TNF-treated NSCs. Cells were treated with TNF for 3 days and analyzed for DNA fragmentation using a TUNEL assay (Fig. 4). DNAse treatment as a positive control resulted in nearly 75% TUNEL-positive nuclei. Label solution as a negative control gave no positive signal. In contrast, some apoptotic nuclei were detected under control conditions. TNF treatment increased the rate of apoptosis in NSCs only moderately (Fig. 4). This result suggests that a major aspect of the physiological action of TNF on NSCs might be the induction of proliferation.

### TNF does not interfere with neural stem cell differentiation

**Figure 5:**
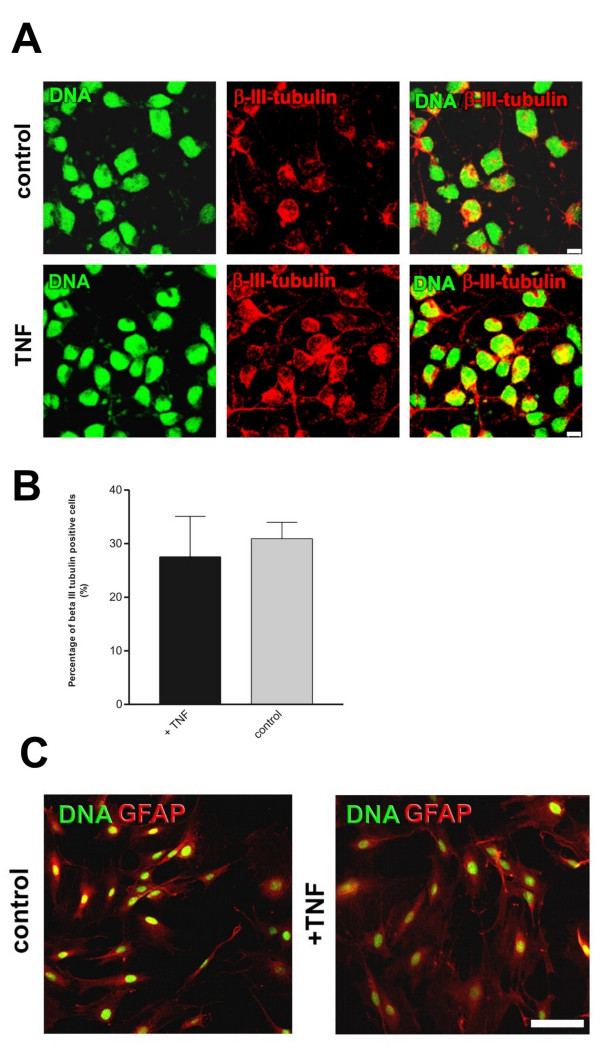
**TNF does not interfere with neural stem cell differentiation**. (A) Immunostaining of neuronally differentiated neurosphere-derived NSCs. Cells were cultured on poly-D-lysin/laminin-coated dishes in the absence of EGF and bFGF, as described in Materials and Methods. β-III-tubulin expression as a marker for immature neurons was up-regulated in the cells 3 days after adhesion to the coated substrate. (B) TNF treatment does not significantly change the percentage of β-III-tubulin positive cells (P > 0.05) Bars depict 5 μm. (C) Dissociated NSCs were cultured for four days in the absence of cytokines in medium containing 10% FCS followed by fixation and staining for GFAP. Note that TNF treatment had no effect on the capacity for differentiation into the glial lineage. Bar, 100 μm.

The crucial property of stem cells is to differentiate into more specialized cells, in our case into neuronal progenitors, immature neurons or glial cells. We therefore investigated whether TNF might influence the capacity of NSCs to differentiate into neurons. TNF-treated and untreated cells were plated on laminin/poly-D-lysine coated culture dishes for up to 3 days. This protocol is frequently used to induce neuronal differentiation of NSCs. Cells positive for β-III tubulin (a marker for immature neurons) with neuron-like morphology were detected 3 days after plating both with and without TNF treatment (Fig. 5A). The frequency with which immature neurons differentiated from the NSCs was not significantly affected (p > 0.05) by TNF treatment (Fig. 5B). In addition, TNF treatment had no effect on the capacity of the cells to differentiate into the glial lineage, as demonstated by immunocytochemical staining for glial fibrillary acidic protein (GFAP) four days after plating in the presence of 10% fetal calf serum (FCS) (Fig. 5C). This suggests that the action of TNF might be specific only for the proliferation of NSCs; cell fate is not affected.

### TNF activates NF-κB in neural stem cells

**Figure 6:**
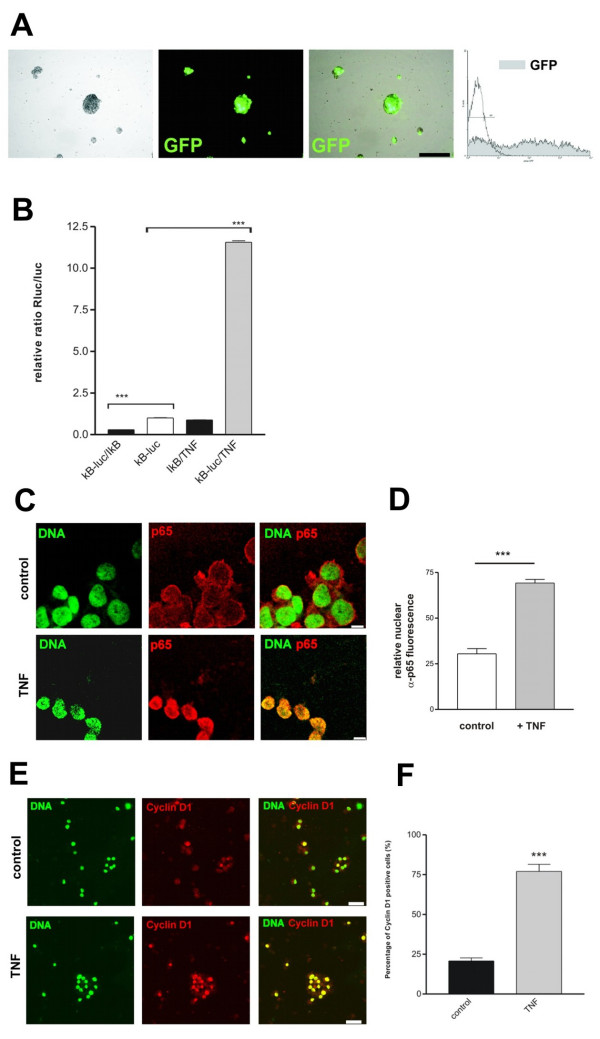
**The effect of TNF on NSCs**. (A) TNF activates NF-κB in NSCs as measured by reporter gene activity and nuclear translocation of the p65 subunit. Transfection efficacy was measured with CMV-driven GFP expression plasmids (upper panel). The left image depicts a phase contrast image of transfected cells. The middle image shows GFP expression. Both images are merged in the right image. Bar depicts 200 μm. Flow cytometry was used to quantify the transfected cells. Control cells (untransfected) are shown in white. Transfected cells are shown in grey. Note the high efficacy of transfection (about 60%). (B) NSCs were transfected using the optimized conditions shown in (A). Induction of NF-κB by TNF was more than ten-fold. Basal κB-dependent reporter gene expression, as measured by bioluminescence under control conditions, was set to 1. (C) TNF-induced nuclear localization of the transactivating NF-κB subunit p65. Nuclei were stained with SYTOX (green). Activity-specific anti-p65 immunoreactivity is depicted in red (middle panel). Merged images are shown on the right. Note the basal NF-κB activity in unstimulated cells (upper panel). Bar depicts 5 μm. (D) Representative fields of cells were analyzed to determine the mean p65 fluorescence in the nucleus. Note that TNF strongly increases nuclear p65 compared to untreated controls. (E) TNF treatment elevates cyclin D1 expression in NSCs. Cold-shock synchronized neurospheres were collected on glass slides by cytospin centrifugation, fixed and stained for cyclin D1. DNA was stained using SYTOX (green). Note the higher cyclin D1 expression in TNF-treated cells (lower panels) compared to controls (upper panels). (F) Quantification of cyclin D1 expression. TNF-treated neurospheres show significantly higher numbers of cyclin D1-positive cells than untreated neurospheres (*** P < 0.001). Bars, 20 μm.

One of the major signal transduction pathways activated by TNF is the canonical NF-κB pathway. To investigate TNF-mediated signal transduction, a highly efficient transfection system was needed. We used a modified Amaxa electroporation protocol (see Materials and Methods), which transfected up to 60–80% of the cells, as shown by fluorescence microscopy and flow cytometry (Fig. 6A). To evaluate the efficacy of transfection, we used a commercially available expression vector for green fluorescent protein (pmaxGFP, Amaxa, Köln, Germany).

In view of the well-described activation of NF-κB by TNF in other systems such as various tumor cell lines, we transfected NSCs with κB-luc reporter vector and Renilla-luc control vector (Dual-Luciferase Reporter Assay System, Promega, Mannheim, Germany) using the method described above. Expression of the NF-κB luciferase reporter plasmid was measured using bioluminescence (Fig. 6B). Basal NF-κB activity in mock-transfected control cultures was set to a relative standard value (v = 1). Co-transfection of the NF-κB reporter plasmid and the NF-κB super-repressor IκB-AA1 significantly decreased the activity measured. In contrast, TNF treatment increased NF-κB activity more than 10-fold compared to untreated controls.

Next, we analyzed the sub-cellular distribution of NF-κB p65. Untransfected NSCs were fixed and immunostained with a p65-antibody (Fig. 6C). This activation-specific antibody can only react with the nuclear localization signal (NLS) of p65 when the repressing IκB protein is not present [27]. TNF treated NSCs showed increased nuclear localization of p65 (Fig. 6C, lower panels) compared to untreated controls (upper panels). Thus, the subcellular localization of NF-κB correlates with the activation.

### TNF treatment elevates expression of cyclin D1 in NSCs

Several reports have shown that the cyclin D family has a crucial role in cell cycle progression, so we hypothesized that cyclin D might also be a bona fide target gene of NF-κB in NSCs. We analyzed the expression of cyclins D1, D2 and D3 in unsynchronized cells and detected strong expression of cyclin D1 in some cells (data not shown). We then synchronized a NSC culture by cold shock (4°C, 24h, see [28] for review). The synchronized cells were brought back to 37°C for 4 h (cell cycle release) and stimulated with TNF. This treatment strongly activated the expression of cyclin D1 (Fig. 6E, F). As expected, cyclin D1 was mainly restricted to the NSC nuclei after TNF treatment (note yellow nuclei in lower panel of Fig. 6E). Cyclin D1-positive cells increased more than 4-fold (Fig. 6F) compared to untreated, synchronized cells. Interestingly, TNF treatment did not change the expression of cyclins D2 and D3 (data not shown).

### NF-κB regulates neural stem cell proliferation

**Figure 7:**
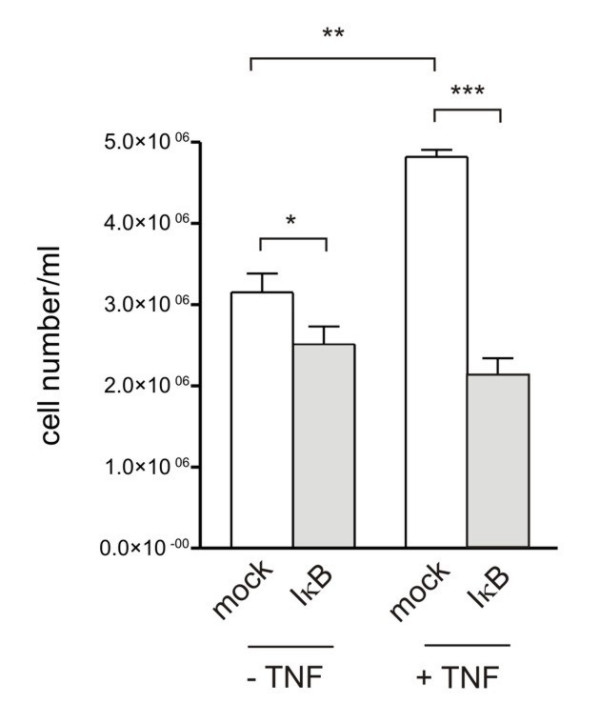
**NF-κB activation is responsible for TNF-induced NSC proliferation**. Note that NF-κB activation is also necessary for the proliferation of NSCs under control conditions (compare mock-transfected control versus IκB transfected cells). Transfected cells without IκB expression vector were stimulated with TNF. Induction of proliferation by TNF is significantly abrogated by IκB co-transfection (***P < 0.001).

Next, a functional assay for the role of NF-κB in NSC proliferation was established using transfection with the transdominant negative super-repressor IκB-AA1. Mock-transfected, unstimulated cells showed basal NF-κB activity, which was decreased by the expression of IκB-AA1 (see Fig. 6B) and resulted in a significantly lower cell number (Fig. 7). When cells were stimulated with TNF for 2 days, proliferation was significantly (p < 0.0001) reduced when the super-repressor IκB-AA1 was transfected (Fig. 7). TNF treatment caused increased proliferation of control NSCs, while the division of IκB-AA1 transfected cells was not significantly affected (p > 0.05). Taken together, these data suggest that NF-κB activation might be responsible for NSC proliferation without the need for additional exogenous TNF (Fig. 7, compare mock-transfected control versus IκB-transfected cells). These data further demonstrate that blockade of the NF-κB pathway via transcriptional repression leads to decreased proliferation of NSCs.

### TAK-1 and IKK-β gene knock down decreases endogenous and TNF-induced proliferation of NSCs

**Figure 8:**
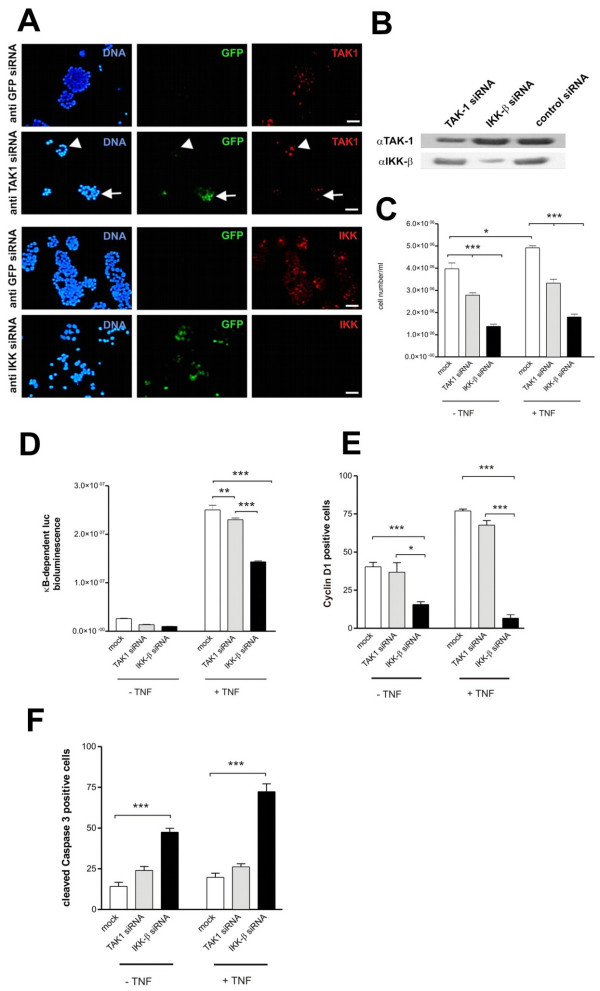
**Gene knock down of TAK-1 and IKK-β impairs TNF-induced stem cell proliferation**. (A) NSCs were co-transfected with siRNA for GFP and GFP expression vector to monitor the efficacy of the knock down strategy (upper panel). After fixation, all cells were stained for TAK-1 and IKK-β and counterstained for DNA with DAPI (blue). Note the expression of TAK-1 (red) and IKK-β in cells transfected with siRNA for GFP (green). In contrast, cells co-transfected with siRNA for TAK-1 (red) and GFP expression vector, or with anti IKK-β siRNA and GFP, expressed minimal levels of knocked down proteins (see arrowheads). Note that non-transfected, GFP-negative cells showed unaffected TAK-1 expression (marked by arrowhead). Bars represent 200 μm. Immunocytochemical results were confirmed by western blotting. Note also the unaffected expression of TAK-1 and IKK-β in control (anti GFP siRNA transfected) cells. (B) Western blot analysis of gene knock down. (C) Quantification of proliferation in siRNA transfected cells. After dissociation of transfected neurospheres, the cell number was determined as described in Materials and Methods. Note the stronger negative effect of IKK-β knock down compared to TAK-1 silencing. (D) Reporter gene assay showed down-regulation of NF-κB activity after gene knock down. NF-κB activity was most markedly inhibited after IKK-β knock down. (E) Analysis of cyclin D1 expression in cells transfected with siRNAs. Note that the most prominent effect was observed in IKK-β siRNA-transfected cells after TNF treatment. (F) Activation of caspase 3 in cells transfected with IKK-β and TAK-1 siRNA. Note the strongest effect of IKK-β knock down after TNF stimulation.

**Figure 9:**
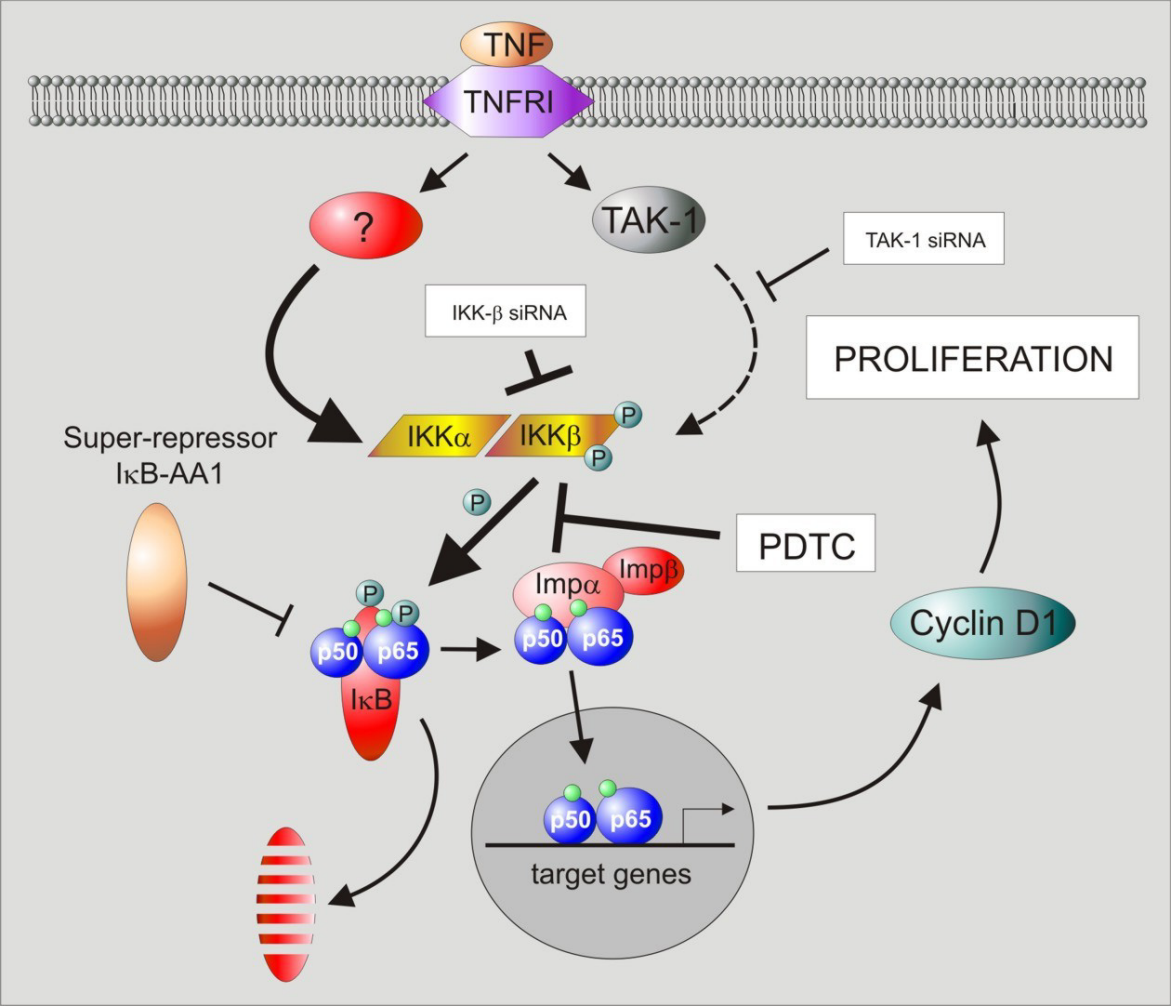
**Model for the TNF-dependent activation of proliferation is NSCs**. The TNF receptor is depicted in purple in the plasma membrane. Activation induces phosphorylation of IκB-α via IKKα/β complex at ser 32 and 36, which subsequently leads to ubiquitination of IκB by IkB ubiquitin ligase and degradation within the proteasome resulting in nuclear translocation of p50/p65. Transcription of NF-κB target genes such as cyclin D1 is responsible for proliferation and can be repressed by expression of the transdominant negative super-repressor IκB-AA1. In addition, pharmacological blockade of IκB ubiquitin ligase or silencing of IKK-β via siRNA leads to strongly decreased cyclin D1 expression and proliferation of NSCs. In contrast, TGFβ activated kinase-1 (TAK-1) seems to be involved but is not crucial for TNF-mediated NF-κB activation.

In view of the well-established role of IKKs in TNF-mediated signaling, we designed a specific siRNA for IKK-β. Some reports have suggested a potential role for TGFβ-activated kinase-1 (TAK-1) in the signal cascade under investigation, suggesting that construction of a siRNA for this kinase could be informative (Fig 8A and 8B). Gene knock down of both TAK-1 and IKK-β led to significantly decreased proliferation in comparison to cells transfected with control (anti-GFP) siRNA. Interestingly, we observed that IKK-β knock down affected proliferation more markedly than the silencing of TAK-1 (see Fig 8C). This general tendency was independent of TNF treatment.

### TAK-1 and IKK-β knock down decrease NF-κB activity

We studied the effect of the siRNAs against TAK-1 and IKK-β on NF-κB activity?. The cells were transiently co-transfected with siRNA for TAK-1 and κB-luc, or with siRNA for IKK-β and κB-luc. Endogenous NF-κB activity was markedly decreased after IKK-β knock down. In contrast, the effect of TAK-1 on NF-κB activity was much weaker (Fig. 8D). TNF treatment led to activation of NF-κB in siRNA-transfected and un-transfected cells. Nevertheless, IKK-β siRNA-transfected cells showed markedly decreased NF-κB activity compared to both untransfected and TAK-1 siRNA transfected cells. Thus, NF-κB activity correlated with the effect on proliferation after knock down of TAK-1 and IKK-β (compare Fig. 8C and 8D).

### IKK-β but not TAK-1 knock down leads to significantly decreased cyclin D1 expression

On the basis of our finding that cyclin D1 was up-regulated after TNF treatment, we investigated the influence of TAK-1 and IKK-β knock down on cyclin D1 expression in NSCs. Silencing of IKK-β resulted in strongly decreased cyclin D1 expression, while TAK-1 knock down induced only a moderate decrease (Fig. 8E).

### IKK-β silencing but not TAK-1 knock down induces apoptosis in NSCs

Finally, we investigated the potential induction of apoptosis by the siRNAs. NSC apoptosis was enhanced by IKK-β knock down (see Fig. 8F). TNF treatment had no significant effect on the cleavage of caspase-3 and therefore on the induction of apoptosis in control or TAK-1 siRNA-transfected cells (Fig. 8F). In contrast, IKK-β siRNA-transfected cells showed a strongly increased apoptosis rate after TNF treatment (Fig. 8F). Because TNF activated apoptosis only moderately in control cells, the up-regulation of caspase-3 cleavage might be explained by a higher apoptosis rate in cells transfected with anti-IKK-β siRNA.

Thus, TAK-1 knock down once again had a weaker biological effect than IKK-β silencing. Overall, IKK-β silencing strongly reduced NF-κB activity and cyclin D1 expression. In addition, proliferation stopped and apoptosis was induced, demonstrating that NF-κB has a dual role in NSCs: survival and proliferation.

## Discussion

In this study, we have described a novel mitogenic action of TNF on adult neural stem cells. We investigated the signaling mechanism involved and showed that proliferation is crucially dependent on the activation of NF-κB. Moreover, we showed for the first time that an active IKKα/β complex and active downstream IκB ubiquitin ligase are essential for NF-κB activation and the resulting proliferation of NSCs, as indicated by the pharmacological blockade of IκB ubiquitin ligase and gene silencing of IKK-β via siRNA. In addition, we showed that TNF treatment induces up-regulation of cyclin D1 expression, and we demonstrated an involvement, but not a crucial role, of TAK-1 in the signal transduction cascade investigated.

In accordance with our data, TNF was recently identified as a mitogen in the subventricular zone of rats. Stereotactical injection of TNF-α into the lateral ventricle resulted in a highly significant increase in proliferating cells 24 h post-injection [29]. Here we have shown that TNF has a novel cell-autonomous action on isolated neural stem cells. This might rule out the potential effects of the endogenous stem cell niche. Several reports have shown a marked up-regulation of TNF expression under pathological conditions such as infection with Gram negative bacteria, Alzheimer's disease, Parkinson's disease and even brain inflammation [11]. On the other hand, Cacci et al. reported that TNF is toxic to neural precursor cells when proliferation has ceased [30]. In contrast, few apoptotic cells were found in the SVZ of TNF-treated animals [29].

There is much evidence for up-regulation of chemokine expression and secretion in situations of brain pathology [11]. Interestingly, most such situations are characterized by TNF secretion into adjacent tissues. In a previous study we showed marked up-regulation of MCP-1 expression and secretion by astroglioma cells after TNF treatment [10]. Subsequently, we identified MCP-1 as a potent chemoattractant for NSCs [13]. It is to be expected that in pathological situations NSCs will migrate from their endogenous niche (SVZ) in response to chemokines such as MCP-1, released at the area of inflammation [11]. Thus, after migration, NSCs are exposed to TNF. In this context, our results suggest that a major aspect of the physiological action of TNF on NSCs might be the induction of proliferation.

Taken together, these data suggest that after CNS injury, TNF-α not only plays a critical role in the development of pathology and inflammation, but could also activate NSC proliferation, which might be a physiological means of repair and neuroprotection. In accordance with this hypothesis, Pluchino et al. showed that during CNS inflammation, NSCs are able to promote neuroprotection [31]. This phenomenon might be explained by secretion of neuroprotective cytokines by NSCs.

We used human TNF-α, which has been shown to activate TNF-RI but not TNF-RII in rat neuronal cultures [32,33]. The cultures used in the present study express both TNF-RI and TNF-RII. Human TNF-α has been identified as neuroprotective in hippocampal neurons [32,34] and cerebellar granule cell precursors [35]. Genetic ablation of TNF-RI (p55) exacerbates traumatic brain injury and is correlated with reduced NF-κB activation [14]. In contrast, TNF-α was cytotoxic to mouse-derived neurospheres and interfered with their formation [36]. These data, which conflict with those obtained with rat NSCs, might be attributed either to species differences or the use of murine TNF, which binds to both TNF receptors. The TNF-RI receptor is thought to be the major NF-κB activating TNFR [16].

In a recent study, Mehrhof et al. convincingly showed genetic evidence for a cell type-restricted requirement for NF-κB in the control of proliferation [37]. Thus, the role of NF-κB in proliferation has to be investigated separately for each cell type. In this study, inhibition of NF-κB resulted in a pronounced reduction of NSC proliferation. In the endogenous niche of NSCs – the subventricular zone and the rostral migratory stream – members of the NF-κB family are expressed in dividing cells [38]. In addition, it has been reported that the IKK-α/β-complex is crucial for TNF-mediated NF-κB activation in other systems [17]. Here we have shown for the first time that IKKs are crucial for NF-κB activation and NSC proliferation.

There is evidence for the involvement of TGFβ activated kinase-1 (TAK-1) in the TNF-induced signaling cascade [18,39]. Here we have shown that TAK-1 is involved in TNF signaling in NSCs but is not crucial for NF-κB activation and the resulting proliferation.

Ferguson et al. showed that cyclin dependent kinase 4 and 6 (CDK4/6) signaling is crucial for neural precursor cell cycle regulation [19]. It is well-established that formation of a complex between CDKs 4 and 6 and cyclin D1 is necessary for cell cycle progression. Interestingly, Guttridge et al. demonstrated that NF-κB controls growth and differentiation through transcriptional regulation of cyclin D1 [22]. Here we report for the first time the TNF-mediated up-regulation of cyclin D1 in NSCs. Cyclin D1 is the bona-fide NF-κB target gene, with mapped and functionally characterized NF-κB binding sites in its promoter [22,40].

The expression of NF-κB subunits and the induction of proliferation might suggest that both pathways are linked *in vivo*. We have shown for the first time that NF-κB activation is essential for neural stem cell proliferation. One might ask if TNF-α can also act as a mitogen in other systems. In fact, TNF-α is a growth factor for Hodgkin's lymphoma, cutaneous T-cell lymphoma and gliomas (see [41] for discussion). Recent data suggest that NF-κB is constitutively activated in most cell lines derived from hematopoietic or solid tumors [42]. Similarly, we and others have shown that NF-κB inhibition results in reduced tumor cell proliferation [21,22](see [43] for review).

Indeed, the requirement for activated NF-κB is not the only similarity between dividing tumor cells and stem cells. Recent evidence suggests that formation of tumors might be restricted to cells with stem cell markers [44]. Our data might be especially relevant to brain tumors such as glioblastomas that contain cancer stem cells [45]. Only a few cells are endowed with the ability to replicate and thus to transfer the tumor phenotype residing within medulloblastomas and astrocytomas. These cancer stem cells derived from brain tumors formed NSCs and were positive for the neural stem cells markers Nestin and CD133 [45].

Understanding the mechanisms that regulate stem cell proliferation might be crucial for future regenerative and anti-tumor medicine.

## Conclusion

This study provides experimental evidence that NF-κB is a crucial regulator of NSC proliferation. In addition to providing novel insights in the mechanisms governing adult stem cell self-renewal, this study might add a cautionary note about the use of anti-cell-proliferative agents in tumor therapy.

## Methods

### Isolation and culture of adult neural stem cells (NSCs)

Adult rats were killed by decapitation. NSCs were isolated from the lateral subventricular zone using visually guided micro-preparation under a dissection microscope (Zeiss, Jena, Germany, magnification 8x [2]). About 3 mm of subventricular tissue was prepared with fresh scalpel blades, isolated with Dumont forceps and collected in ice-cold HBSS (Gibco, Eggenstein, Germany) containing 300 mg/ml D-glucose (Sigma, Deisenhofen, Germany). Cells from adjacent tissue not containing stem cells generally do not proliferate in growth-promoting medium. Isolated tissue was digested at 37°C with 1.33 mg/ml trypsin (Sigma, Deisenhofen, Germany), 0.7 mg/ml hyaluronidase (Sigma, Deisenhofen, Germany), 200 U/ml DNAse (Sigma, Deisenhofen, Germany) and 0.2 mg/ml kynurenic acid (Sigma, Deisenhofen, Germany) to dissociate cells. The tissue was passed through a 70 μm cell strainer (BD Falcon; Heidelberg, Germany) and transferred to ice-cold, serum-free medium containing BSA (Sigma, Deisenhofen, Germany) to stop trypsin activity. NSCs were cultured in serum-free medium containing basic fibroblast growth factor (bFGF; 10 ng/ml, Chemicon, Hofheim, Germany), epidermal growth factor (EGF; 20 ng/ml; R&D Systems, Wiesbaden, Germany) and B27 supplement (Gibco, Eggenstein, Germany). Primary neurospheres were dissociated at day 8–10 using Accutase (PAA, Pasching, Austria) to derive clonal neurospheres. The sub-culturing protocol consisted of neurosphere passaging every 3–4 days with whole culture medium change (fresh growth factors were added).

### Immunocytochemistry

Neurospheres were harvested on microscope slides by cytospin centrifugation (212 g, 5 min., Shandon, Thermo, Dreieich, Germany), fixed in 3.7% PFA for 60 min at 4°C and washed 3x in 1x PBS for 5 min. Blocking was performed in 5% appropriate serum for 30 min followed by incubation with anti-Nestin (1:100, BD Pharmingen, Heidelberg, Germany); anti-GFAP (BD Pharmingen, Heidelberg, Germany, 1:100), anti-β-III-tubulin (Promega, Mannheim, Germany, 1:50), anti-TNF-RI (abcam, Cambridge, UK, 1:100), anti-TNF-RII (Alexis Biochemicals, Grünberg, Germany), anti-LeX (Developmental Hybridoma Bank, Iowa City, USA, 1:100), anti Sox2 (Sigma, Deisenhofen, Germany, 1:100), anti-Musashi (Chemicon, 1:100), anti-L1 (Developmental Hybridoma Bank, Iowa City, USA, 1:100), anti-PSA-NCAM (Miltenyi Biotec, Bergisch Gladbach, Germany), anti-Notch1 (Developmental Hybridoma Bank, Iowa City, USA, 1:100), anti-Notch2 (Santa Cruz, 1:50), anti-A2B5 (Chemicon, 1:100), and anti DCX (Santa Cruz, 1:50), anti-TAK-1 (Santa Cruz, 1:50), anti IKK-β (Biosource, 1:50) and anti-cleaved caspase 3 (Cell Signaling Technology, Danvers, USA). Positive antibody binding was detected using a Cy3-conjugated antibody (1:300, Jackson Immuno Research Laboratories, distributed by Dianova, Hamburg, Germany). Nuclei were stained with SYTOX (1:10000, Molecular Probes, Göttingen, Germany). Staining was visualized using confocal laser scanning microscopy (LSM Pascal, Zeiss, Jena, Germany).

### Reverse transcription polymerase chain reaction

Total RNA was isolated (RNAeasy, Qiagen, Hilden, Germany) from adult neurospheres according to the manufacturer's instruction and reverse transcribed. To exclude DNA contamination, mRNA was treated with DNAse (Qiagen). The PCR primers (Qiagen) were: 5'- ATGGGTCTCCCCATCGTGCCTG, 3'- TTATCGCGGGAGGTGGGTCGTG for TNF-RI and 5'- CACACAGTGCCCGCCAAGGTTGT, 3'-TCAAGGCACTTTGACAGCAATCTGGTC for TNF-RII.

### Analysis of neurosphere growth

Secondary neurospheres were harvested, dissociated using Accutase (PAA, Pasching, Austria) and passed through a 40 μm cell strainer (BD Falcon, Heidelberg) to obtain a single neural stem cell suspension. Cells were cultured in serum-free medium containing basic fibroblast growth factor (bFGF; 20 ng/ml Chemicon, Hofheim, Germany), epidermal growth factor (EGF; 20 ng/ml; R&D Systems, Wiesbaden, Germany) and B27 supplement (Gibco, Eggenstein, Germany) and 10 ng/ml TNF (Calbiochem, Schwalbach, Germany). Control cells were cultured in neurosphere medium without TNF. The volume of living neurospheres was measured using an inverse microscope (Axiovert 100, Carl Zeiss, Jena, Germany) and ImageJ software (National Institute of Health, USA; [46]). Statistical significance was determined by ANOVA with Bonferroni correction, using GraphPad's Prism. P ≤ 0.05 was considered significant.

### BrdU incorporation assay

BrdU assays were performed as described [47]. Briefly, 10 μM bromodeoxyuridin (BrdU, Sigma, Deisenhofen, Germany) was added to neurosphere cultures each day. Seventy-two hours after TNF stimulation (10 ng/ml), the neurospheres were harvested on microscope slides by Cytospin centrifugation (212 g, 5 min., Shandon, Thermo, Dreieich, Germany). The cells were fixed with 3.7% PFA for 60 min at 4°C and counterstained with 0.3 μM ToPro-3-iodite (Molecular Probes, Göttingen, Germany). Incorporation of BrdU into newly-synthesized DNA in the NSCs was detected by BrdU-dependent fluorescence enhancement of ToPro-3-iodite. Fluorescence was monitoring using an inverse confocal laser scanning microscope (LSM 5, Pascal, Carl Zeiss, Jena, Germany). Relative cell proliferation was calculated from the fluorescence of 5 fields of view (n = 4 for each condition) as follows: relative percentage of cell proliferatio = (F_exp _- F_min_)/(F_max _- F_min_) × 100. F_exp _is the fluorescence of the experimental test condition, F_max _is the maximal fluorescence and F_min _is the background fluorescence. Differences in relative cell proliferation were assessed by two-way ANOVA followed by a post-hoc t-test with Bonferroni correction. Differences between two conditions at P ≤ 0.05 were considered statistically significant.

### Apoptosis assay

Neurospheres were prepared, cultured and collected as described above, with either 10 ng/ml TNF or neurosphere medium alone. After fixation, an In Situ Cell Death Detection Kit FITC (Roche, Mannheim, Germany) was used for immunocytochemical detection of apoptosis, based on labeling of DNA strand breaks (TUNEL technology) according to the manufacturer's instructions. Fluorescein isothiocyanate (FITC) fluorescence was monitored using an inverse confocal laser scanning microscope (LSM 5, Pascal, Carl Zeiss, Jena, Germany). As positive control, neurospheres were treated with DNAse (2000 U/ml, Sigma, Deisenhofen, Germany). As negative control, labeling solution containing FITC was used. The relative percentage of apoptotic cells was calculated from the fluorescence of 4 fields of view (n = 4 for each condition, relative percentage of apoptotic cells = (F_exp _- F_min_)/(F_max _- F_min_) × 100; key as above). Statistical significance was determined by ANOVA. P ≤ 0.05 was considered significant.

### Determination of cell number

Neural stem cells were plated at 1.0 × 10^6 ^cells/ml in triplicate for each condition. For pharmacological blockade of the NF-κB pathway, neural stem cells were pre-treated for 30 min with 0.1 mM pyrrolidine dithiocarbamate (PDTC, Sigma, Deisenhofen, Germany). TNF-α was added to the cultures at plating. Spheres were collected at 6, 24, 48 and 72 h after plating and dissociated, and total cell numbers were counted. Results were expressed as the mean ± SEM. Statistical significance was determined using two-way ANOVA followed by a post-hoc t-test with Bonferroni correction. Differences between two conditions at P = 0.05 were considered statistically significant.

### Neuronal differentiation of TNF-treated neurosphere-derived cells

Secondary neurospheres were harvested and dissociated as described above. NSCs were cultured in serum-free medium containing B27 supplement and TNF but without EGF and bFGF or in medium without TNF addition and plated on poly-D-lysine/laminin-coated culture slides (BD Biocoat, Heidelberg, Germany). Three days after the TNF stimulus the cells were fixed, stained and analyzed as described above.

### Glial differentiation

Secondary neurospheres were harvested and dissociated, followed by culture in DMEM/F:12 (Gibco) and 10% FBS (PAA, Pasching, Austria). After 4 days of culture the cells were fixed, stained and analyzed as described above.

### Reporter gene assay (transfection and analysis)

Neurospheres were dissociated as described above and transfected using a Rat NSC Nucleofector Kit (Amaxa, Köln, Germany) according to the manufacturer's instructions with minor modifications. In particular, 5.0 × 10^6 ^cells were used for each transfection. After dissociation of the neurospheres (see above), cells were centrifuged at 210 × g for 10 min and re-suspended in an appropriate volume of Amaxa Nucleofector solution. After addition of DNA, the cells were electroporated in an Amaxa device and collected in 10 ml preheated cytokine-free media. After further centrifugation at 210 × g (10min), the cells were re-suspended at appropriate density in cytokine-containing medium. Transfection efficiency was measured using the pmaxGFP vector (Amaxa) and analysis by fluorescence microscopy (Axiovert 100, Carl Zeiss, Jena) and flow cytometry (FACScalibur, Becton Dickinson, Heidelberg, Germany).

To detect NF-κB activity in TNF-stimulated versus unstimulated neural stem cells, a Dual-Luciferase Reporter Assay System (Promega, Mannheim, Germany) and κB-luc Reporter (BD Clonetech, Heidelberg, Germany) was used. All cells were transfected with κB-luc reporter vector and Renilla-luc control vector (Promega, Mannheim, Germany). IκB-AA1 (super-repressor of NF-κB, cloned into the commercially available Rc/CMV expression vector (Promega) [48]) was cotransfected. As a mock vector, pMETalpha (Invitrogen) without insert was used. Forty-eight hours after transfection, the cells were lysed and assayed for promoter-dependent luciferase activity versus promoter independent Renilla-luc activity (Lumat LB9507 device, Berthold Technologies, Bad Wildbach, Germany). A representative experiment is shown.

### Detection of nuclear NF-κB in TNF-stimulated neural stem cells

Neurospheres were stimulated with 10 ng/ml TNF. Thirty minutes after the TNF stimulus, the cells were harvested and fixed as described above. After 30 min permeabilization with 0.5% TritonX100/PBS and blocking with 5% goat serum, the cells were stained with anti-p65 antibody (Chemicon, Hofheim, Germany, 1:50). Nuclear NF-κB was detected with Cy3-conjugated anti-mouse antibody (1:300, Jackson Immuno Research Laboratories, distributed by Dianova, Hamburg, Germany). Nuclei were stained with SYTOX (1:10000, Molecular Probes, Göttingen, Germany). Antibody staining was visualized using confocal laser scanning microscopy (LSM Pascal, Zeiss, Jena, Germany). Laser power and detector settings were kept constant to maintain consistency in the data collection system. Fluorescence intensity was quantified using Image J image analysis software [46]. Statistical significance was determined by ANOVA with Bonferroni correction, using GraphPad's Prism. P < 0.05 was considered significant.

### Detection of TNF-induced cyclin expression

Neural stem cells were dissociated, cultured for 24 h as described above and synchronized by cold shock (24 h, 4°C). After 4 h equilibration at 37°C (release into cell cycle), 10 ng/ml TNF-α was added.

Four hours after TNF stimulation, neurospheres were harvested on microscope slides by Cytospin centrifugation (212 g, 5 min, Shandon, Thermo, Dreieich, Germany), fixed in 3.7% PFA for 60 min at 4°C and washed 3x in 1x PBS for 5 min followed by permeabilization with 0.1% Triton X-100 for 30 min. Blocking was done in 5% appropriate serum for 30 min followed by incubation with anti-cyclin D1 (Sigma, Deisenhofen, Germany, 1:100), anti-cyclin D2 (abcam, Cambridge, UK 1:100) and anti-cyclin D3 (Sigma, Deisenhofen, Germany, 1:100). Antibody binding was detected with Cy3-conjugated antibody (1:300, Jackson Immuno Research Laboratories, distributed by Dianova, Hamburg, Germany). Nuclei were stained with SYTOX (1:10000, Molecular Probes, Göttingen, Germany). Antibody staining was visualized using confocal laser scanning microscopy (LSM Pascal, Zeiss, Jena, Germany).

### Gene silencing (siRNA design and transfection)

The Ambion Silencer siRNA Construction Kit (Ambion) was used to produce siRNAs against TAK-1 and IKK-β. The target sequences were identified and appropriate oligonucleotides constructed as per the manufacturer's instructions. The target sequences for TAK-1 and IKK-β knock down were identified, followed by Blast searches to ensure that the sequences did not contain significant homology to any other known genes. The sequences were TGGCTTATCTTACACTGGA for TAK-1 and GGTGGAAGAGGTGGTGAGC for IKK-β. Both produced similar levels of specific gene product knockdown. In addition, siRNA for GFP (Amaxa, Köln, Germany) was used as a control.

Cells were transiently co-transfected as described above, either with siRNA for the TAK-1 and CMV driven GFP or with siRNA for IKK-β and CMV-GFP, to monitor the efficacy of transfection. All cells were counterstained with DAPI. As control, anti-GFP siRNA and CMV-GFP were transfected. After 48 h, the down-regulation of TAK-1 and IKK-β was analyzed using fluorescence microscopy (Axiovert, Zeiss, Jena, Germany) and western blots.

### Western blot analysis

Soluble cell protein (250 μg per condition) was separated by SDS-PAGE using 8% polyacrylamide gels and electroblotted on to PVDF membranes (Millipore Corporation, Bedford, MA, USA). The membranes were blocked in TBST+3% nonfat dry milk. The following first antibodies were used: mouse monoclonal anti-IKK-β (Biosource) and goat polyclonal anti-TAK-1 (Santa Cruz Biotechnology). For detection, horseradish peroxidase-conjugated goat anti-mouse (Bio-Rad Laboratories GmbH, Muenchen, Germany) and donkey anti-goat (Santa Cruz Biotechnology) IgGs were used with an ECL kit (Amersham Pharmacia Biotech) according to the manufacturer's protocol.

### Influence of gene silencing on proliferation, NF-κB activity, cyclin D1 expression and apoptosis

Anti TAK-1 and anti IKK-β siRNA transfected adult neural stem cells were cultivated for 24 h. Cells were then stimulated with 10 ng/ml TNF and cultivated for up to 48 h. The total cell number was determined as described above.

To investigate NF-κB activity, the cells were co-transfected with siRNA for TAK-1 and κB-luc reporter vector or siRNA for IKK-β and κB-luc. Twenty-four hours after transfection, the cells were stimulated with 10 ng/ml TNF and cultivated for an additional 24 h, then lysed and assayed for promoter-dependent luciferase activity as described above. To study the influence of gene product knock down on cyclin D1 expression and apoptosis, the cells were transfected with siRNAs for TAK-1 and IKK-β followed by 24 h cultivation. Cells were then stimulated with TNF, cultured for an additional 24 h, stained for cyclin D1 and cleaved caspase3 (Cell Signaling Technologies, Danvers, USA, 1:100) and counterstained with DAPI. GFP-positive cells were analyzed for TAK-1 and IKK-β immunoreactivity.

## Abbreviations

BrdU Bromo-deoxy-uridine

CDK4/6 cyclin dependent kinase 4 and 6

CNS central nervous system

IKK IkappaB kinase

IκB-α IkappaB-alpha

MCP-1 Macrophage Chemoattractant Protein 1

NF-κB nuclear factor kappa B

NPC neuronal progenitor cells

NSC neural stem cells

PDTC pyrrollidine dithiocarbamate

SCF stem cell factor

SDF1 stromal derived factor 1

siRNA short interfering RNA

SVZ subventricular zone

TAK-1 TGFβ activated kinase 1

TNF-R Tumor necrosis factor receptor

TNF-α Tumor necrosis factor-alpha

VEGF vascular endothelial growth factor

## Competing interests

The author(s) declare that they have no competing interests.

## Authors' contributions

DW performed the NSC isolation, cell culture, analysis of proliferation, reporter gene assays, transfections and immunocytochemistry; IM performed RT-PCR and Western Blot analysis; CK contributed to project conception and experimental design and wrote the draft of the manuscript; BK contributed the siRNA expression vectors, and conceived and supervised the study. ME performed a part of the TUNEL assay. All authors read and approved the final manuscript.
